# Can we predict firms’ innovativeness? The identification of innovation performers in an Italian region through a supervised learning approach

**DOI:** 10.1371/journal.pone.0218175

**Published:** 2019-06-11

**Authors:** Ilaria Gandin, Claudio Cozza

**Affiliations:** 1 Research Unit, Area Science Park, Trieste, Italy; 2 Department of Economic and Legal Studies—DISEG, University of Naples “Parthenope”, Naples, Italy; Universita degli Studi di Foggia, ITALY

## Abstract

The study shows the feasibility of predicting firms’ expenditures in innovation, as reported in the Community Innovation Survey, applying a supervised machine-learning approach on a sample of Italian firms. Using an integrated dataset of administrative records and balance sheet data, designed to include all informative variables related to innovation but also easily accessible for most of the cohort, random forest algorithm is implemented to obtain a classification model aimed to identify firms that are potential innovation performers. The performance of the classifier, estimated in terms of AUC, is 0.794. Although innovation investments do not always result in patenting, the model is able to identify 71.92% of firms with patents. More encouraging results emerge from the analysis of the inner working of the model: predictors identified as most important—such as firm size, sector belonging and investment in intangible assets—confirm previous findings of literature, but in a completely different framework. The outcomes of this study are considered relevant for both economic analysts, because it demonstrates the potential of data-driven models for understanding the nature of innovation behaviour, and practitioners, such as policymakers or venture capitalists, who can benefit by evidence-based tools in the decision-making process.

## Introduction

Although technological change has been considered as a key driver of economic growth by classical economists, its measurement has always been problematic. Even a more precise conceptualisation of technological innovation in firms, as put forward by Schumpeter [1] who firstly distinguished between product and process innovations, has not solved this issue. One of the main reasons resides in the heterogeneity and complexity of the innovation process. Either considering it as a linear or a chain-linked model [2], an analyst is first intrinsically asked to declare if its measurement is focusing on the input, output or the specific behaviour of firms engaged in the innovation process; and then, concerning firms’ behaviour, whether an object or subject approach is to be followed [3]. Such a variety in the range of possible measurement of innovativeness is reflected in two major strands of empirical studies, a first one relying on the use of patents and a second one on innovation surveys. These two sets of data sources have been largely exploited in both qualitative and quantitative terms, especially relying on inferential statistics and econometrics. Only recently, pioneering studies have started using machine learning approaches with a particular reference to innovation process. We believe this novel methodology can help better exploiting these types of data.

### Patents as measures of innovation

Over the last decades, the quantitative and more objective measurement of innovation output (mostly patents and scientific publications) has made significant steps forward, even more than in the case of innovation input (Research and Development, R&D). However, some of these measures are not considered adequate for assessing innovation in all types of firms, especially in terms of economic sectors and firm size. The typical example of such a high variability is represented by the patenting propensity, especially if compared to firms’ R&D efforts: “While the propensity to patent differs significantly across industries, the relationship between R&D and patents is close to proportional, especially for firms above a minimal size. Small firms do receive a significantly higher number of patents per R&D dollar but this can be explained by their being a much more highly selected group” [4]. There is also another degree of variability, that cannot be linked to any “typology” (size, sector etc.) of firms but is intrinsically connected to their individual behaviour: “Not all inventions are patented. Firms sometimes protect their innovations with alternative methods, notably industrial secrecy” [5]. Additionally, the use of patents raises the problem of the assessment of their “value”, either considering it as a “scientific” or “economic” value. No conclusive results have been found, but over the last decades very different and controversial proxies of this innovation measurement have been proposed: use of backward and forward citations; family size; number of claims; impact on the launch of innovative products or on the overall performance of patenting firms (within a very large literature, we highlight the conceptualisations put forward in: Harhoff et al. [6], Reitzig [7,8], Hall et al. [9] and Messeni Petruzzelli et al. [10]).

### The Community innovation survey

To overcome this limitation in using patents, since early 1990s many studies dealing with the direct measure of innovation in Europe have relied on the outcomes of the Community Innovation Survey (CIS) and to its statistical guidelines contained in the Oslo Manual [11]. This is a clear example of a subject-approach to innovation measurement: with the CIS, in fact, a sample of firms in each EU country is asked to report a set of answers on their innovation behaviour, usually every two years. Studies using data from the CIS have been constantly growing since its first launch [12]. The reasons behind this use have also evolved over time and the richness of CIS data that have been gathered made it possible to answer very different research questions. In particular, the focus has shifted from the determinants of innovation internal to the firm, to “open innovation” strategies [13]. Just to make few recent examples, Leiponen [14] has used CIS to study the difference in R&D and external knowledge sourcing as the determinants of manufacturing *vs*. service innovation; Ghisetti et al. [15] to test the effect of knowledge sourcing on environmental innovations; Ardito & Messeni Petruzzelli [16] to assess the moderating role of human resource practices on the relation between knowledge sourcing and product innovation.

However, also CIS data have limitations, mostly related to their high statistical costs. In fact, CIS has not a census approach, at least not for all firms. As a result, the main problem in CIS questionnaires concerns the distribution of observations by firm size. In fact:

- CIS has a census approach only for large firms (those with more than 250 employees);
- it has a sample and rotation approach for small and medium enterprises (SMEs, that is with 10 to 249 employees);
- it does not cover micro firms (with less than 10 employees), that are therefore excluded from this type of analysis.

As a result, especially in those EU countries/regions where a very large majority of firms is represented by SMEs and micro firms, it is likely that information is often missing about firms’ innovation behaviour. This might have an impact on a subject willing to know whether a specific firm is innovative or not. It might be the case of a policymaker asking for ex-ante measures to optimally allocate the resources of an innovation policy or its ex-post evaluation; or the case of a venture capitalist who needs information to support an investment decision. In addition to the issue of size distribution, CIS is meant for statistical purposes, thus implying its confidentiality. Both a policymaker or a venture capitalist are not allowed to explicitly know which firms are innovative or not.

### Machine learning approach

The investigation on these issues could gain a new prospective with machine learning techniques, which have shown great potential in the context of resource allocation problems in complex settings. Machine learning algorithms have the capacity to learn rules (i.e. models) from sample data that can be put in practice to make prediction on new data. Such methodologies are suitable when the amount of data is “big” in both dimensions: when data is “wide”, because the number of variable possibly involved is large, making difficult to form assumptions on the underlying relationships, and when the number of observations is “long”, ensuring sufficient informative content for the model to come to light. Thanks to the advances in digitalisation of administrative data and processes, relevant data sources have become widely available in the public sector that can be directed to feed data-driven algorithms providing valuable support for policy and decision problems. Successful examples can be found in health management, urban development, economic growth, educational system, public inspections [17,18]. The key idea is that machine learning algorithms provide empirical evidence to inform policy. Prediction methods offer a unique support for the evaluation of different type of intervention-actions, their applications and relative benefit/cost ratio. Moreover, being data-driven, models selected through machine learning algorithm usually show higher performance compared to other approaches. Such information, together with context-specific constraints and targets, are the crucial elements to be considered in the decision process that administrators experience in complex problems related to resource management. In economic studies, a very similar framework has been successfully employed for the target group selection in marketing decision problems [19]. Models are used to estimate response probabilities in marketing campaigns so that they can be restricted to likely responders. However, applications of machine learning algorithms, even in more general settings, are still concentrated in few economic sub-sectors (e.g. in finance; see Mullainathan and Spiess [20] for a recent survey) and only some recent studies have dealt with the economics of innovation and firm behaviour. Among them, Hajek et al. [21] take Europeans NUTS 2 regions as units of observation and apply multi-output neural networks to model innovation performance. Considering instead micro-level analyses, Noh et al. [22] focus on the forecasting of patent citations; while Skute et al. [23] on the composition of university spin-offs. To the best of our knowledge, however, no study deals with the research question we present in this paper: is it possible to predict firms’ innovativeness? Thanks to the availability of a wide variety of data sources that can be integrated and mutually enriched, the analysis of this problem seems nowadays not only feasible but also appropriate, since any step forward in detecting innovation propensity in firms can have a big impact on the development of better and more efficient investment policies. We face this question in the remainder of this paper: first we briefly review the literature on the drivers of innovation propensity; then we describe the data chosen for the analysis and the methodology employed; following sections report the results obtained and a discussion of results; the last section concludes.

## Materials and methods

### Innovation propensity and its drivers in economic literature

In line with the objectives depicted above, we rely on the extensive literature on the drivers of firms’ innovativeness to identify one specific measure of innovation (as dependent variable) and a set of explanatory variables. Concerning the dependent variable, we consider innovative those firms having reported innovation expenditures in a reference year, as it is common in the CIS. An alternative might be that of using the more qualitative questions available in the CIS, that is having introduced any kind of innovation in the reference period; eventually, a breakdown of innovativeness can derive from the type of innovation introduced (product, process, organizational or marketing innovation). However, we have opted for the use of innovation expenditures for three reasons: it can be considered the more “objective” and “quantifiable” variable in the CIS; being a monetary variable, its use is more convenient as almost all explanatory variables are monetary as well; economic literature [24] has clearly highlighted that the type of innovation introduced should be linked to the type (internal *vs*. external) sources of information, that are available only for a subset of firms in our sample.

We will deal now with explanatory variables. Since the seminal contributions of Griliches [25] and Griliches [4], the introduction of the concept of “knowledge production function” has given origin to many theoretical and empirical studies on the links between R&D, innovation and productivity, employing three- or four-equations models [26–29]. Micro-level studies have also explored the case of Italy, using one or more waves of the Italian CIS, whose sub-sample will be analysed in this paper. These works (e.g. [30–35]) confirm and adapt to the Italian case results obtained in other countries’ analyses, thus implying that results for this country are generically representative of innovation in industrialised countries. In all these contributions, the innovation equation is modelled as a function of R&D investment and (directly or indirectly) of firms’ past performance, controlling for size, sector and location of firms. In the work by Crepon et al. [27], innovation is mostly driven by R&D that, in turn, is explained by firm size, demand pull and technology push factors. Further improvements of this model have progressively introduced additional variables (e.g. ICT expenditures in Hall et al [34]). Bogliacino et al. [35] model “innovation expenditures” as a function of previous innovation expenditures, turnover and labour compensation. Rather often, both firm-level and meso-level (sectoral) studies have tested alternative variables in terms of economic performance. For instance, Bogliacino et al. [36] include lagged profits as a measure of internal financial resources. They follow the idea by Hall [37] that financing constraints are key for R&D and innovation investments: this implies considering all explanatory variables such as tax incentives, retained earnings (or profits), debt and new shares (or venture capital in the case of start-ups).

Since none of these models can be considered conclusive of the R&D-innovation-productivity link, and given the data-driven approach that has been adopted in our analysis, the paper starts with a very wide inclusion of explanatory variables from those studies, putting together firms’ structure and balance sheet information to predict their innovation behaviour. In line with the supervised-learning framework, the functional form of the model, explanatory variables (or “predictors”), as well as possible non-linear interactions between predictors, are determined as a function of the data, with the aim of maximising the goodness of fit. The only limitation that has been applied on the inclusion of variables is related to their availability: since we are interested in the potential innovativeness of all firms in a sample, we need to rely on a set of variables that are generally available. Therefore we use balance sheet variables not only because of their use in economic literature, but also as they are potentially available for all the firms we want to study in terms of prediction.

### Data

The analysis in this study is performed on the cohort of firms with at least 10 employees and resident in the Italian NUT2 region “Friuli-Venezia Giulia”. Although smaller in terms of population and regional GDP as compared to other Northern Italian regions, Friuli-Venezia Giulia constantly ranks high for innovation in Italy, according to the EU regional Innovation Scoreboard [38]. It is a region characterised by a long-term industrial development and also scientific excellence, thus an appropriate case study for our analysis.

The main sources of data concerning innovation performance are the ISTAT CIS 2012 and 2014 questionnaires, though they target only a sample of FVG firms. Even if innovation behaviour can be complex and heterogeneous, it can be encoded into a binary dependent variable equal to 1 (positive innovation propensity) whenever a firm reports some innovation expenditures, and 0 if innovation expenditures are null (negative innovation propensity). Overall, these innovation expenditures include: R&D intra muros, R&D extra muros, acquisition of machinery and software, know-how, planning and design, training, marketing and other preliminary innovation activities. Thus, this study will propose a classification model assigning firms to either innovators class (INN) or non-innovator class (N-INN). Since R&D expenditures are considered in CIS as a subset of total innovation expenditures, additional observations are added to the study extracting information on firms’ investments from R&D questionnaires (ISTAT RS1 survey for year 2012 and 2014). Positive innovation propensity is inferred for firms that claim positive expenditure for intra muros R&D OR extra muros R&D. It is reasonable to assume that a company claiming R&D expenditure in the RS1 questionnaire would have reported those expenditures into the CIS survey too.

Given the nature of this research, great effort has been put to collect detailed firm-level data on the different domains potentially related with innovation behaviour. The starting point for the definition of the cohort is the InfoCamere registry, supervised by the Italian Chamber of Commerce. In addition to the basic identification data, InfoCamere registry provides full-digits primary NACE codes that are classified based on the two main taxonomies strongly related to innovation. The first is the Eurostat technological intensity classification that distinguishes high-tech (HT), med-high-tech (MHT), med-low-tech (MLT), low-tech (LT), knowledge-intensive service (KIS) and less knowledge-intensive service (LKIS) firms. The second is a revisited Pavitt Taxonomy [39] classification that is more focused on technological sources and on the diversity of appropriability regimes across sectors. It classifies both manufacturing and service companies into science-based operators, specialised suppliers, scale and information-intensive operators, suppliers dominated operators. Moreover, the number of employees is obtained from the ISTAT registry of active firms (ASIA) and integrated in the dataset.

Another set of independent variables are selected from the balance sheet and profit&loss account items as provided by ORBIS database (Bureau van Dijk Electronic Publishing). In this context, the study focuses on firm size, structure of fixed assets, current assets, liabilities and profitability ratios. For a detailed list of variables see Table 1. Standard quality control procedures are applied to quantitative variables: in case of outlier or inconsistent values (showing vulnerable situations) the firm is removed from the sample. In order to exclude very critical insolvency situations (that can distort innovation propensity), firms are excluded if their leverage exceeds 15.55 (the 90th percentile). Moreover, firms with consolidated financial statement are dropped since unconsolidated data could not be found. In order to make the results easier to be interpreted, strongly correlated variables (Pearson correlation coefficient ρ > 0.5) are excluded from the input variables. Given the predictive framework of the study, financial statement refers to the previous year with respect to the innovation survey.

**Table 1:** Data availability for years 2011 and 2013. (N.A.-Not Available).

Variable	Available 2011	N.A. 2011	Available 2013	N.A. 2013	Note
InfoCamere entry	2975	0	2975	0	-
No. of employees	2944	31	2934	41	> = 10
Pavitt class	2853	122	2904	71	-
Technological class	2853	122	2904	71	-
Turnover	2384	591	2786	189	log−transf.
Turnover on cost of employees	2370	605	2755	220	log−transf.
Profit−loss on cost of employees	2370	605	2755	220	-
Share of intangibles on fixed assets	2382	593	2491	484	sqrt−transf.
Share of tangibles on fixed assets	2382	593	2491	484	sqrt−transf.
Share of fixed assets on total assets	2382	591	2493	482	sqrt−transf.
Creditors turnover ratio	2379	596	2493	482	log−transf.
Employee average cost	2360	615	2762	213	log−transf.
ROS	2370	605	2490	485	-
ROI	2375	600	2490	485	-
ROE	2384	591	2493	482	-
Leverage	2384	591	2493	482	< = 15.55 excluded
Net long term debt	2378	597	2488	487	log−transf.
Net short term debt	2378	597	2488	487	log−transf.

Information on patent filing is collected from the EPO Worldwide Patent Statistical Database (PATSTAT) to provide further evidence on model predictions. For each firm, the number of patents deposited from 2011 to 2015 is extracted. In the analysis, innovation class is compared to the number of patents of the two-years period next to the survey wave (2012–2013 for 2012 survey, 2014–2015 for 2014 survey).

### Methodology

The study implements a predictive framework: 1) a classification model based on companies’ features is calculated for survey respondents (survey sample) 2) the obtained model is applied to the remaining companies (out-of-survey firms) to predict their class. Bearing in mind the heterogeneity across firms within the cohort, which may implicate complex interactions among variables, the classification problem is approached with the random forest algorithm [40]. Random forest is an ensemble method based on decision trees: a large collection of trees is built on bootstrapped sub-samples and then results are averaged over the trees. More specifically, the algorithm retrieves a class vote from each tree, so that each observation obtains a large number of votes (depending on bootstrapping selection). Votes can be then expressed as proportions on the total number and in case of binary classification, as a value in [0,1] (class probability). A good feature of random forest is that for each tree, observations discarded because of the bootstrapping—referred as Out-Of-Bag (OOB) data—can be used to perform an internal validation and contribute to obtain a global estimate of the error (OOB error estimate).

In this study random forest is calculated using the default hyper-parameters of the RandomForest R package [41]. The number of features randomly sampled (“mtry”) is the square root of the total (rounded down), minimum size of terminal nodes is 5, the number of trees is 500, splitting criteria is Gini importance. For each observation, the result of the model is a class probability estimate in [0,1] that is obtained averaging the votes of all trees forming the forest. Model’s performance is measured based on the mean squared error (MSE) of the assigned class probabilities. Since no model calibration is performed, in order to fully exploit the sample size, the model is calculated on the whole sample and its performance on unseen data (model checking) is evaluated with a 10-fold cross-validation (CV) built on the same dataset [42,43]. K-fold CV is a good choice for model checking when data is scarce. Indeed, working sample is partitioned in k equal-sized parts to mimic the classic resampling method based on train-test split: for each fold, a surrogate model is trained on the remaining (k − 1)-folds and the fold itself is used as test set to calculate the surrogate MSE. Then, the mean of the k-surrogate models’ errors gives the cross-validation MSE estimate (CVMSE).

Random forest can be considered a black-box algorithm, since no direct information is given about the relationship between predictors and the outcome variable. In order to gain some insights into the underlying mechanism, predictors are analysed from two points of view. First, their importance in terms of contribution to predictive accuracy (mean decrease accuracy) is reported. Secondly, their impact on the predicted outcome is investigated through partial dependent plots (PDPs) and individual conditional expectation (ICE) plots. The idea of PDPs is to focus on one independent variable at a time, say the i-th variable, and analyse the behaviour of a function approximating its marginal distribution. Such relationship is estimated over the training data averaging the value of the model as a function of i-th variable [44]. ICE plots can be considered a refinement of PDPs since instead of an average curve, a group of curves are graphed for individual observations [45]. Together, PDPs and ICE plots highlight the global trend as well as possible heterogeneous effects.

After model definition, the decision rule problem is analysed: the output generated by the model is a class probability and the problem requires the assignment to either INN or N-INN group. Therefore a decision threshold (i.e. the operating point) within [0,1] should be set and used to split observations into the two classes. The chosen value influences the discriminatory ability of the model. First, the analysis of Receiver Operating Characteristic (ROC) curve is performed to visualise the balance between sensitivity (the fraction of true positives among INN class) and specificity (the fraction of true negatives among N-INN). Based on ROC analysis, the operating point is defined according to the characteristics of the classification problem and the context of its application.

## Results

The cohort consists of 2975 firms. After the exclusions for missing data (Table 1) and quality control procedures, analysis is restricted to 2688 firms including 935 respondents to at least one of the mentioned surveys. The study focuses on firms’ features, therefore survey answers of the two waves (2012 and 2014) form a unique sample. Given the census approach for large companies, 323 firms have records for both 2012 and 2014. Multiple answers of the same company referring to different years are considered as independent records because they are probably influenced by profound changes occurring within the time gap. As a consequence, the working sample for model computation counts 1258 observations (Table 2). Variable distributions across subgroups (observations grouped by survey wave and survey type) are extensively investigated showing no bias (see Tables A and B in S1 Table). After an appropriate transformation of strongly skewed variables (logarithmic/square-root transformation), analysis of correlation identifies few strongly correlated variables (see Table 3). Based on that, number of employees, tangibles, added value per employee, net long-term debt and ROI are discarded from the analysis, restricting the model to 13 independent variables.

**Table 2:** Number of observations suitable for the analysis with all necessary data available. Unit of observation “firms” refers to the count of firms, while “answers” refers to the number of records of the surveys.

Unit of observation	CIS	R&S	No survey	Tot
Firms	873	62	1753	2688
Answers	1145	113	2580	3838

**Table 3:** Correlated variables.

Var1	Var2	Roh
log−Turnover	log−No. emp	0.84
sqrt−Intangibles	sqrt−Tangibles	−0.61
log−Turnover	Added value/empl	0.51
P−L/cost empl	Added value/empl	0.63
Added value/empl	log−Empl avg	0.57
log−Short debt	log−Long debt	0.76
P−L/cost empl	ROI	0.55
ROI	ROS	0.58

Random forest algorithm is applied on the whole survey sample with default parameters (resultant “mtry” is 3). Thanks to the internal bootstrapping, random forest makes possible to exploit OOB observation to obtain an unbiased estimate of the prediction error. The obtained model shows OOB MSE equal to 0.183. In order to have a more robust estimate, 10-fold cross-validation is performed on the same sample. The CVMSE is 0.184, thus substantially confirming the OOB estimate.

**Fig 1:**
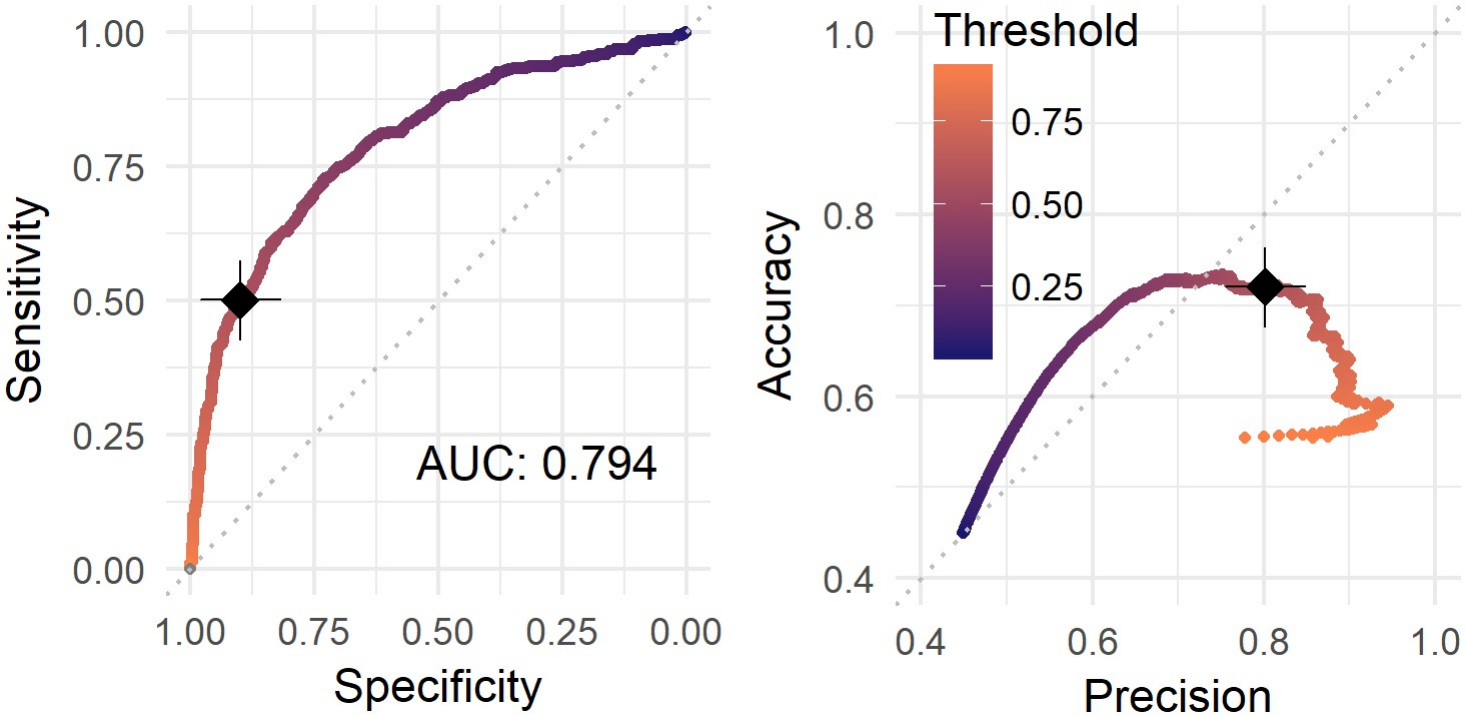
On the left, Receiver Operating Characteristic (ROC) curve. On the right, precision-accuracy curve. A black cross has been placed next to the chosen threshold value 0.589.

Classes are slightly unbalanced: INN represent the 44.91% of the sample and N-INN the 55.09%. The baseline performance for a classification model is 55.09% of accuracy and 50% of precision. As for other target-group selection problems, the desirable model should perform well in terms of precision (i.e. the proportion of true positive among those firms predicted as innovative). The rationale behind this is the preference for a selected group of firms very likely to be innovative, instead of a larger group with less performing results. In order to analyse the effect of different decision thresholds (operating points), the shape of the ROC curve on OOB votes is investigated. The curve is obtained considering a dense sequence of possible thresholds within the response range that splits observations into the two classes. For each threshold, specificity and sensitivity are calculated. The area under the curve (AUC), which is a global indicator of performance, measures 0.794 (Fig 1). The common choice for decision threshold, as the closest point to [1,1] (in our case, threshold = 0.436), would lead to a precision of 68.73%, which is unlikely suitable for target selection problems. Instead, accuracy-precision trade-off (Fig 1) suggests threshold = 0.589 as a good choice aiming to obtain 80.17% of precision, 72.02% of accuracy, 89.90% of specificity and 50.09% of sensitivity.

**Fig 2:**
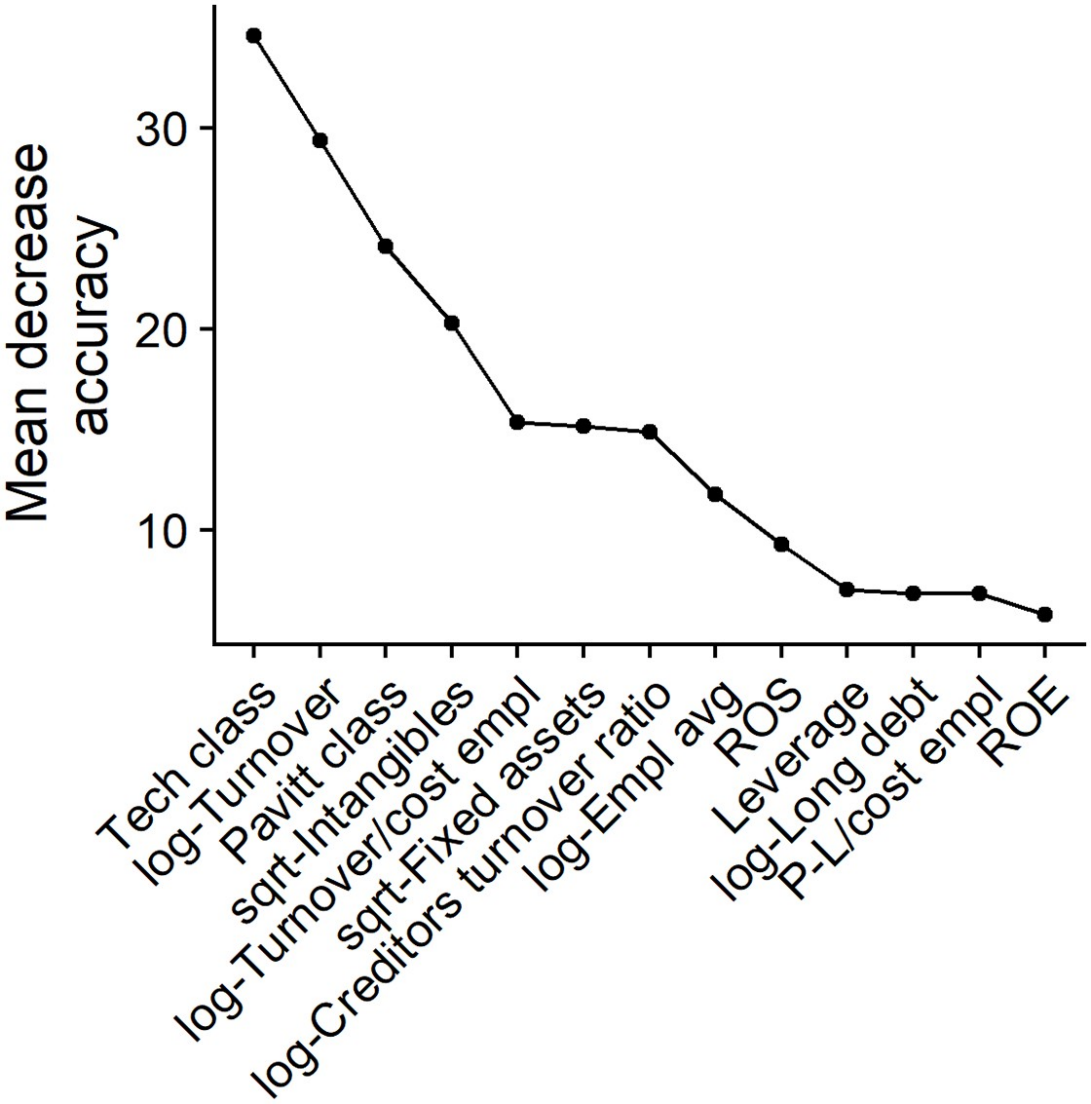
On the first axis, variable names. On the second axis, variable importance measured as the mean decrease in accuracy.

In Fig 2 variables are ranked by mean decrease in accuracy which is obtained permuting the values of each variable and measuring how much the permutation decreases the classification accuracy. Using the elbow rule, 4 most important factors can be identified: technological class, firm size (based on turnover), Pavitt class and structure of fixed assets (share on intangibles).

**Fig 3:**
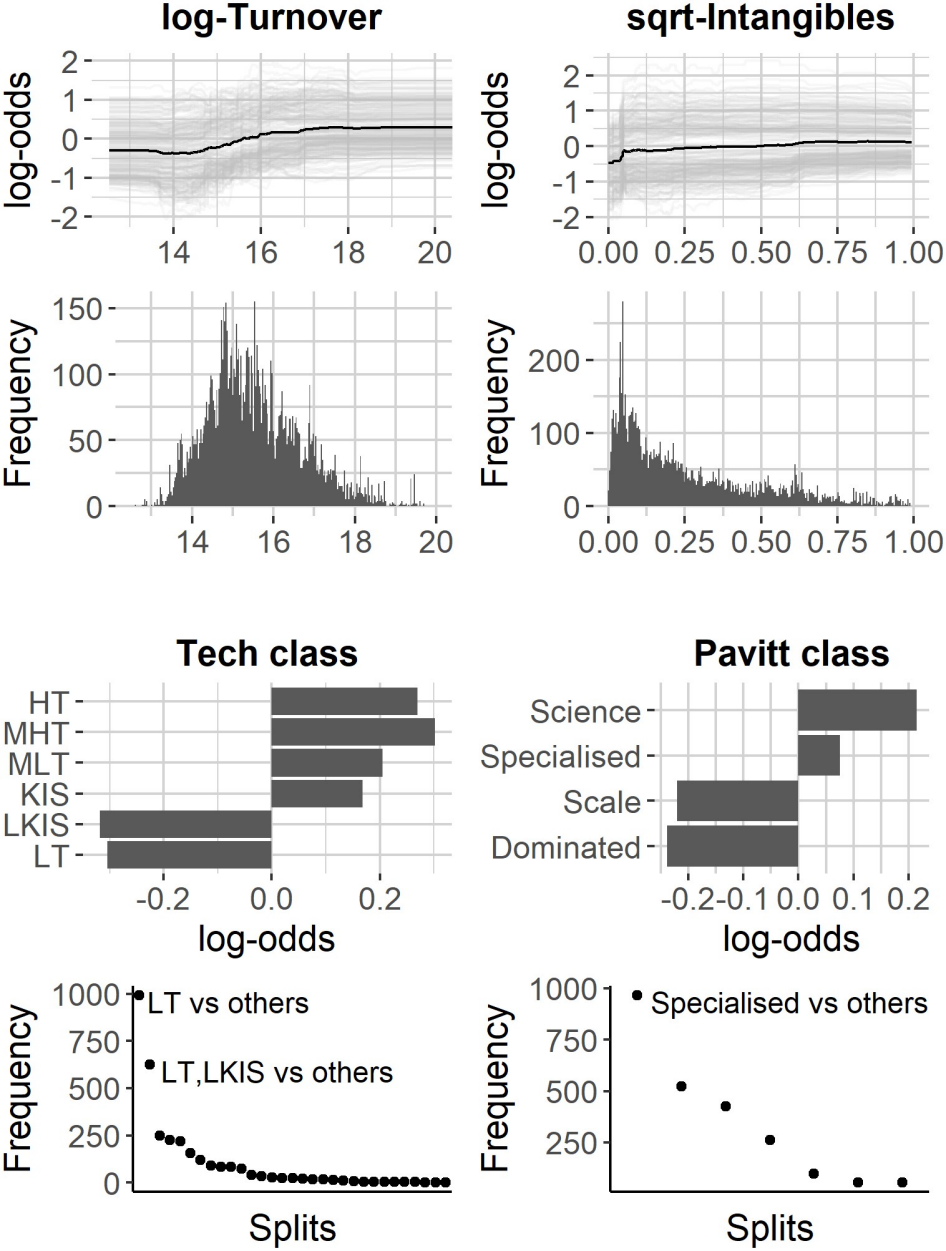
On the top: PDP and ICE plot for continuous variables. On the bottom: PDP for categorical variable. For turnover and intangibles, the 20% of conditional expectation curves (randomly chosen) are reported in light grey while the average curve is marked in dark-grey. The aligned histogram plot represents the distribution of the splitting value used to build the trees. For technological and Pavitt class a simplified PDP is shown together with the most frequent splits.

Another way to gain some insights on the black-box of the algorithm is to visualise the average behaviour of the outcome response function for each feature. In Fig 3 the result of PDP and ICE plot analysis are reported for the most important quantitative variables: turnover and intangibles. For each variable, we consider the curves given by the functional relationship between the variable itself and the change in log-odds of the prediction. The graphs highlight different contribution for the two variables. In both cases there is a substantial positive monotonic contribution to probability for class INN. However, in the case of intangibles the increase is concentrated in a small range of values suggesting the presence of a threshold effect. This fact is confirmed by the distribution of the splitting values used by the algorithm. In random forest, once a variable is selected, an optimal splitting value is chosen to add a new bifurcation on the tree. Depending on the initial conditions (sampling of features and observations), such value varies and its distribution can be analysed. The histogram of splitting values in Fig 3 confirms that for intangibles there is a hot-spot of splitting values around 0.002 corresponding to the threshold identified through ICE plots.

For categorical variables (technological class and Pavitt class) the analysis is similar. PDP simply considers the average value of the model for each of the variable classes (see Fig 3). Also in this case, it is interesting to analyse PDPs together with the frequency of binary splits. For technological class, two highly frequent branches can be observed: the first one splits observations between LT *vs*. all the others, while the second one groups LT and LKIS *vs*. the others. Both LT and LKIS show a negative effect for INN class. For Pavitt classes there is one exceptionally frequent split that distinguishes between specialised suppliers *vs*. all the rest, where being a specialised supplier increases the chances for the INN class.

Finally, the model is applied to the remaining 2580 out-of-survey observations (as for the survey sample, data over two years): 715 (28.57%) are assigned to class INN, thus predicted as innovative.

An interesting comparison between model predictions and innovation outputs can be done considering information about patent filing. For each observation, the number of patents deposited within the survey year (2012 and 2014) is considered. Even if the absolute numbers are very small, it is interesting to compare model predictions and companies having any patent either to the Italian Patent Office (UIBM) or directly to the European patent office (EPO). As displayed in Table 4, among the 57 observations that have at least one patent, 41 of them (71.9%) are actually predicted by the model as innovative (in survey sample, where true labels are known, the proportion is 92%). From another point of view, it can be appreciated a substantial difference of patenting rate between classes: within the N-INN group, only 0.9% (16 out of 1865) of observations have a patent for the reference year, while for the INN group the percentage is 5.7% (41 out of 175).

**Table 4:** Number of patents for N-INN and INN groups. First row refers to firms with at least one UIBM patent; second row to firms with at least one EPO patent; third row to firms with at least one patent (either UIBM or EPO).

Variable	N-INN	INN	N-INN prop.	INN prop.
UIBM	9	29	0.237	0.763
EPO	7	15	0.318	0.682
any	16	41	0.281	0.719

To sum up, the major findings of this study are:

- the development of a predictive model having performance 0.794 measured in terms of AUC;
- the identification of technological class, turnover, sectorial class and intangibles as most important predictors of innovation;
- the classification of out-of-survey observations and the identification of an INN*-predicted* group that includes 71.9% of the patenting firms.

## Discussion

As shown in Fig 2 and commented above, among the 13 explanatory variables employed in our methodology, four variables are identified as key for prediction accuracy. It is not a surprise that they reflect firms’ structural characteristics rather than their economic or financial behaviour in the period antecedent to innovation expenditures. Sectoral classifications (in our case, “Tech class” and “Pavitt class”) and firm size (in our case, “log of Turnover”) are always accounted in literature as control variables in estimations having innovation as dependent variable. In other words, it is always expected that belonging to specific groups of sectors (e.g. high-tech vs. low-tech) or size classes (large firms vs. SMEs) would explain, at least partially, firms’ innovation behaviour. Our results, although employing a predictive and not an inferential methodology, yield to the same point. Even the last key explanatory variable—that is the share of intangible assets over total fixed assets—although directly coming from balance sheet data, can be considered as a structural variable. It is unlikely, in fact, that firms change suddenly their fixed assets from more tangible to more intangible: as other studies have demonstrated [46,47], our findings confirm that the share of investment in intangibles explains firms’ innovation behaviour.

However, our results provide further insights, as shown in Fig 3. Concerning continuous variables ("log of Turnover” and “Intangibles”), they behave differently: the association of turnover with innovation expenditure has a smooth shape. Vice versa, the intangibles variable seems to have a threshold value, also particularly low. This might mean that, in order to increase the probability of innovate, firms should have any intangible assets in their balance sheet.

Let us turn now to categorical variables, that is sector taxonomies. In both cases, there is one or two splits selected by the algorithm more frequently than the other ones. Concerning the technological taxonomy, it is key for the innovation propensity that firms do (not) belong to lower technological categories: low-tech manufacturing or less-knowledge intensive services. This is quite obvious. However, the association of variables is even stronger if there is a split between low-tech manufacturing only and all the rest of categories. In other words, sectors belonging to less technological services appear to be less hampered in innovation than in the case of manufacturing. Given the longer tradition of North-Eastern Italian firms (especially SMEs) in low-tech manufacturing, this might show that these ones face higher difficulty in investing in innovation rather than service firms, that are probably younger, albeit not knowledge intensive. However, this result might be highly dependent on the definition of innovativeness we have adopted in this paper, that is innovation expenditure as from the CIS questionnaire. Indeed, the innovativeness of low-tech firms might be linked to less measurable strategies, to the development of the (national or regional) innovation systems in which they are embedded and to the tacit knowledge dynamics of their employees [48–50]. When looking at the revised Pavitt taxonomy variable, the key split is the one dividing between Specialised Suppliers sector and all the other ones. Also this result is in line with previous studies, as we refer to smaller firms that have to invest in technological innovation in order to survive in more and more competitive global value chains, often with large and demanding actors (e.g. multinationals) as clients.

Finally, Table 4 shows the application of the model to firms out-of-survey, that is firms that have not revealed their innovation (or R&D) behaviour through statistical questionnaire over the considered period. Going back to what anticipated in the introduction, it is interesting to test the quality of our prediction using another type of indicator: patent applications, either to the Italian or to the European Patent Office. Despite the limitations related to the use of patents as measure of innovation, already recalled in the introduction, they are still the most widely used variable in literature and the unique benchmark we can use to test our prediction. Results in Table 4 show that the prediction algorithm correctly identifies as innovative a share of firms out-of-survey (71.9%) not too distant from the survey sample (92%).

## Conclusions

In this study a supervised machine learning approach has been proposed to investigate the innovation propensity of a sample of firms based in an Italian region. The first step has been a proper codification of variables involved: given that explanatory factors were selected from financial statements, as they are available in principle for all firms, also the outcome variable representing innovation propensity has been codified in monetary terms. Thus, in this study innovation propensity means having innovation expenditures in a given year. As a second step, based on the characteristics of the problem, random forest algorithm has been chosen to calculate the predictive model. The efficacy of the prediction has been largely studied with cross-validation and relative performance metrics. Since benchmarks of performance for this problem could not be found in literature, model predictions have also been analysed and compared with patenting information. Moreover, to unravel the black-box mechanism underlying the model, predictors effects have been investigated giving useful information on variable importance and interpretation.

The major limit of this study is the performance of the model which reaches high precision only shifting the operating point: we do not look for a continuous value of innovation propensity, but we try to identify those firms that are likely to make innovation expenditures. Analysis of variables suggests a possible reason for that: the proposed model represents a macro-level segmentation of firms and it is scarcely sensitive to the specific innovation attitude that may distinguish firms with common structural features. Although such results might appear trivial, they can nevertheless be meaningful to (regional) policymakers. They are invited to constantly pay attention to the structural features of industries in their territories, in terms of economic sectors of activity, technological intensity and firm size. This attention is usually high when performing ex post evaluation, often relying on inferential statistics. We claim here that machine learning methodologies might instead be important for ex ante evaluations: in order to increase the innovation potential of their regions, policymakers are suggested to promote a structural change of their territories. In other terms, they might use machine learning techniques to define policies that are coherent with the innovation potential of their regions (countries). In order to go beyond these limitations, a strategy to detect a more firm-specific behaviour could be that of including the variations of financial variables across years as predictors. However, such framework clearly leads to a higher level of heterogeneity that cannot be handled with the current sample size. As a consequence, the most relevant direction for future research is the replication of the analysis with the same data at the national, or even international, level. We believe that, analysing larger cohorts, additional differentiations across firms would emerge, leading to the possible identification of novel explanatory factors of innovation.

Another limitation of the study is the exclusion of micro firms—those with less than 10 employees—that are a large majority in many countries, and definitely in Italian regions. However, this limitation is intrinsically linked to the research strategy of using variables from the CIS questionnaire as outcome variables. Indeed, this type of survey across EU countries is always excluding firms under a certain firm size. From this point of view, even the replication of the analysis at the national or international level would face the same problem.

Despite the limitations highlighted, still the proposed framework seems to be promising. Explanatory factors confirm what found in previous studies and, for the first time, they are used in a predictive study. As a result, the objective proposed in the introduction—giving a policymaker or an investor the possibility to know in advance whether a specific firm is innovative or not—is found to be feasible. This is an important message in times when both public and private supporters of innovative firms and start-ups request more evidence-based instruments.

## Supporting information

S1 Table **A. Distribution of the variables of interest separately for years 2011 and 2013.** For both years, first quartile, median, mean and third quartile are reported. Welch’s t-test is used to compare the means of the two distributions (significance level α = 0.01) and the relative p-value is reported in the last column. None of the variables shows a significant difference. **B. Distribution of the variables of interest separately for firms claiming positive expenditures for R&D in CIS and those claiming same type of expenditure in R&D survey.** A significant difference can be found for turnover (and consequently for turnover / cost of employees). This difference is due to the different approach adopted for small and medium firms (sampling approach for CIS and census approach on potential R&D performers for R&D survey) and thus other subtle and potentially problematic bias can be excluded.
(PDF)
