# Supplementary material for: Can we predict firms’ innovativeness? The identification of innovation performers in an Italian region through a supervised learning approach
Source: PLoS One. 2019 Jun 11;14(6):e0218175. doi: 10.1371/journal.pone.0218175 (PMC6559647; doi:10.1371/journal.pone.0218175)
Supplement: S1 Table — A. Distribution of the variables of interest separately for years 2011 and 2013. For both years, first quartile, median, mean and third quartile are reported. Welch’s t-test is used to compare the means of the two distributions (significance level α = 0.01) and the relative p-value is reported in the last column. None of the variables shows a significant difference. B. Distribution of the variables of interest separately for firms claiming positive expenditures for R&D in CIS and those claiming same type of expenditure in R&D survey. A significant difference can be found for turnover (and consequently for turnover / cost of employees). This difference is due to the different approach adopted for small and medium firms (sampling approach for CIS and census approach on potential R&D performers for R&D survey) and thus other subtle and potentially problematic bias can be excluded. (PDF) [file pone.0218175.s001.pdf]

Supporting information

Table A.

Variable	1.Qu 2011	Med 2011	Mean 2011	3.Qu 2011	1.Qu 2013	Med 2013	Mean 2013	3.Qu 2013	p-val
**log-Turnover**	14.53	15.20	15.46	16.19	14.54	15.36	15.59	16.41	8.9e-02
**log-Turnover/ cost empl**	1.21	1.56	1.67	2.09	1.18	1.53	1.65	2.06	7.2e−01
**P-L/cost empl**	0.00	0.06	0.15	0.24	0.01	0.05	0.14	0.20	9.0e−01
**sqrt-Intangibles**	0.06	0.15	0.23	0.34	0.07	0.15	0.25	0.37	1.5e−01
**sqrt-Tangibles**	0.82	0.96	0.87	0.99	0.78	0.95	0.85	0.99	1.6e−01
**sqrt-Fixed assets**	0.36	0.52	0.51	0.67	0.36	0.52	0.51	0.66	8.8e−01
**log-Creditors turnover ratio**	0.10	0.16	0.16	0.21	0.10	0.15	0.15	0.20	8.7e−02
**log-Empl avg**	10.32	10.51	10.49	10.67	10.33	10.52	10.51	10.70	1.1e−01
**ROS**	0.04	0.07	0.09	0.13	0.04	0.07	0.09	0.12	9.2e−01
**ROI**	0.04	0.08	0.10	0.14	0.05	0.08	0.10	0.13	6.8e−01
**ROE**	0.06	0.16	0.23	0.38	0.06	0.16	0.22	0.34	4.3e−01
**Leverage**	0.30	1.00	0.95	1.66	0.18	0.82	0.85	1.62	7.4e−02
**log-Long debt**	0.00	0.32	1.23	1.88	0.00	0.37	1.23	1.75	9.8e−01
**log-Short debt**	0.00	0.96	1.73	2.95	0.00	0.81	1.72	2.95	9.0e−01

Table B.

Variable	1.Qu CIS	Med CIS	Mean CIS	3.Qu CIS	1.Qu R&S	Med R&S	Mean R&S	3.Qu R&S	p-val
**log-Turnover**	15.05	16.29	16.34	17.33	15.06	15.68	15.68	15.75	3.0e−04
**log-Turnover/ cost empl**	1.26	1.62	1.69	2.09	1.29	1.50	1.50	1.55	1.9e−02
**P-L/cost empl**	0.00	0.07	0.17	0.29	0.01	0.05	0.05	0.11	1.6e−01
**sqrt-Intangibles**	0.10	0.24	0.32	0.52	0.12	0.26	0.26	0.34	5.3e−01
**sqrt-Tangibles**	0.72	0.89	0.81	0.98	0.72	0.90	0.90	0.81	7.6e−01
**sqrt-Fixed assets**	0.40	0.57	0.54	0.68	0.39	0.57	0.57	0.55	5.2e−01
**log-Creditors turnover ratio**	0.13	0.17	0.18	0.22	0.12	0.16	0.16	0,17	3.3e−01
**log-Empl avg**	10.43	10.61	10.58	10.79	10.39	10.59	10.59	10.56	4.1e−01
**ROS**	0.05	0.09	0.11	0.13	0.06	0.09	0.09	0.10	7.7e−01
**ROI**	0.06	0.09	0.10	0.14	0.05	0.09	0.09	0.11	8.7e−01
**ROE**	0.06	0.16	0.20	0.31	0.06	0.16	0.16	0.18	6.9e−01
**Leverage**	0.20	0.91	0.87	1.60	0.24	0.87	0.87	0.85	9.1e−01
**log-Long debt**	0.00	0.60	70.09	4.53	0.00	1.02	1.02	96.96	6.9e−01
**log-Short debt**	0.09	1.98	82.66	19.01	0.07	1.80	1.80	188.59	4.4e−01
